# Cholinergic modulation enables scalable action selection learning in a computational model of the striatum

**DOI:** 10.1038/s41598-025-18776-3

**Published:** 2025-10-07

**Authors:** Álvaro González-Redondo, Jesús A. Garrido, Jeanette Hellgren Kotaleski, Sten Grillner, Eduardo Ros

**Affiliations:** 1 Department of Computer Engineering, Automation and Robotics, Information and Communications Technology Research Center of the University of Granada (CITIC-UGR), Granada, Spain; 2 Department of Neuroscience, Karolinska Institutet, Stockholm, Sweden; 3 Science for Life Laboratory, Department of Computer Science, KTH Royal Institute of Technology, Stockholm, Sweden

**Keywords:** Dopamine, Acetylcholine, Spiking Neural Network, Spike-Timing-Dependent Plasticity, Neuromodulation, Reinforcement Learning, Computational neuroscience, Learning algorithms, Motor control, Basal ganglia, Synaptic plasticity, Spike-timing-dependent plasticity

## Abstract

The striatum plays a central role in action selection and reinforcement learning, integrating cortical inputs with dopaminergic signals encoding reward prediction errors. While dopamine modulates synaptic plasticity underlying value learning, the mechanisms that enable selective reinforcement of behaviorally relevant stimulus-action associations–the structural credit assignment problem–remain poorly understood, especially in environments with multiple competing stimuli and actions. Here, we present a computational model in which acetylcholine (ACh), released by striatal cholinergic interneurons, acts as a channel-specific gating signal that restricts plasticity to brief temporal windows following action execution. The model implements a biologically plausible three-factor learning rule requiring presynaptic activity, postsynaptic depolarization, and phasic dopamine, with plasticity gated by cholinergic pauses that temporally align with behaviorally relevant events. This mechanism ensures that only synapses involved in the selected behavior are eligible for modification. Through systematic evaluation across tasks with distractors and contingency reversals, we show that ACh-gated learning promotes synaptic specificity, suppresses cross-channel interference, and yields increasingly competitive performance relative to Q-learning in complex tasks, reflecting the scalability of the proposed learning mechanism. Moreover, the model reveals distinct roles for striatal pathways: direct pathway (D1) neurons maintain stimulus-specific responses, while indirect pathway (D2) neurons are progressively recruited to suppress outdated associations during policy adaptation. These findings provide a mechanistic account of how coordinated cholinergic and dopaminergic signaling can support scalable and efficient reinforcement learning in the striatum, consistent with experimental observations of pathway-specific plasticity.

## Introduction

Learning which actions lead to rewarding outcomes is a core challenge in natural environments, where multiple cues and competing alternatives coexist. Reinforcement learning (RL) primarily addresses value learning–estimating expected rewards^1^–but faces challenges in credit assignment, particularly when outcomes are delayed or when multiple stimuli compete for behavioral relevance. Efficient learning thus requires mechanisms that can selectively reinforce the relevant associations while ignoring irrelevant ones, a phenomenon known as cue competition or structural credit assignment^2^.

We distinguish between two forms of credit assignment: *temporal credit assignment* concerns identifying which past events led to current outcomes across time delays, while *structural credit assignment* involves determining which inputs, among many simultaneous candidates, are causally responsible for outcomes^2^. While basic RL algorithms naturally handle immediate structural assignment, they struggle with temporal credit assignment without additional mechanisms. Importantly, when multiple stimuli are presented with temporal gaps (as with distractors), structural credit assignment inherently becomes a temporal problem.

The basal ganglia, particularly the striatum, are central to RL by integrating cortical representations of context and action with dopaminergic reward prediction error (RPE) signals^3^. Synaptic plasticity in the striatum is modulated not only by dopamine (DA)^4–7^, but also by acetylcholine (ACh)^8–10^, released by cholinergic interneurons (CINs). CINs fire tonically but exhibit characteristic pause-rebound patterns in response to salient events^11,12^. These pauses, often coinciding with DA bursts, define temporal windows that gate plasticity, requiring the coincidence of cholinergic pause, phasic DA, and postsynaptic activity^9^. This gating may help restrict synaptic change to behaviorally relevant inputs, addressing cue competition and enhancing specificity^9^ (for mechanistic details, see the Supplementary Note).

While early views considered CIN pauses to be global^12^, recent findings show spatial and functional heterogeneity across striatal regions^13^, with pause patterns depending on reward, movement, and context^14–20^. This suggests a potential mechanism for channel-specific modulation: CIN pauses restricted to the striatal region activated during a successful action could enable localized plasticity (see the Supplementary Note for detailed biological rationale). We hypothesize that this form of action-specific cholinergic modulation implements a solution to the classical structural credit assignment problem by preventing interference from irrelevant channels. In our computational implementation, we abstract this complex thalamo-striatal-cholinergic circuitry through simplified “action neurons” that trigger both behavioral actions and local cholinergic gating signals, without explicitly modeling CINs or their tonic firing patterns (see Methods for details).

Here we present a computational model in which ACh acts as a channel-specific gating signal–*i.e*., it enables plasticity only in the striatal subpopulation associated with the executed action. The model implements a three-factor learning rule (presynaptic activity, postsynaptic depolarization, phasic DA), but restricts plasticity to periods of local cholinergic pauses. Compared to models without ACh gating, our model improves learning speed and synaptic specificity. It also enhances adaptability in tasks with distractors and contingency reversals. Additionally, the model reproduces functionally distinct roles for D1 and D2 pathways, consistent with experimental observations^21–23^, with D1 SPNs maintaining stimulus-response associations and D2 SPNs facilitating behavioral adaptation^24,25^. This mechanism improves learning efficiency in the tasks considered here as task complexity increases and offers a potential solution to structural credit assignment^15,26–28^.

## Methods

### Behavioral tasks

We designed a set of behavioral tasks in which agents learn to associate specific stimuli with correct actions through trial and error. Each stimulus activates a distinct temporal pattern of synthetic but biologically inspired neural activity, mimicking structured dynamics observed in cortical and hippocampal regions^29,30^, which project to the striatum^31^. In the basic task, each trial presents a single stimulus and requires selecting the corresponding action to receive a reward. In the credit assignment task, multiple stimuli are presented simultaneously–only one is behaviorally relevant–requiring the model to resolve sensory ambiguity. In reversal tasks, stimulus-action contingencies are remapped during training to test the model’s flexibility.

**Fig. 1:**
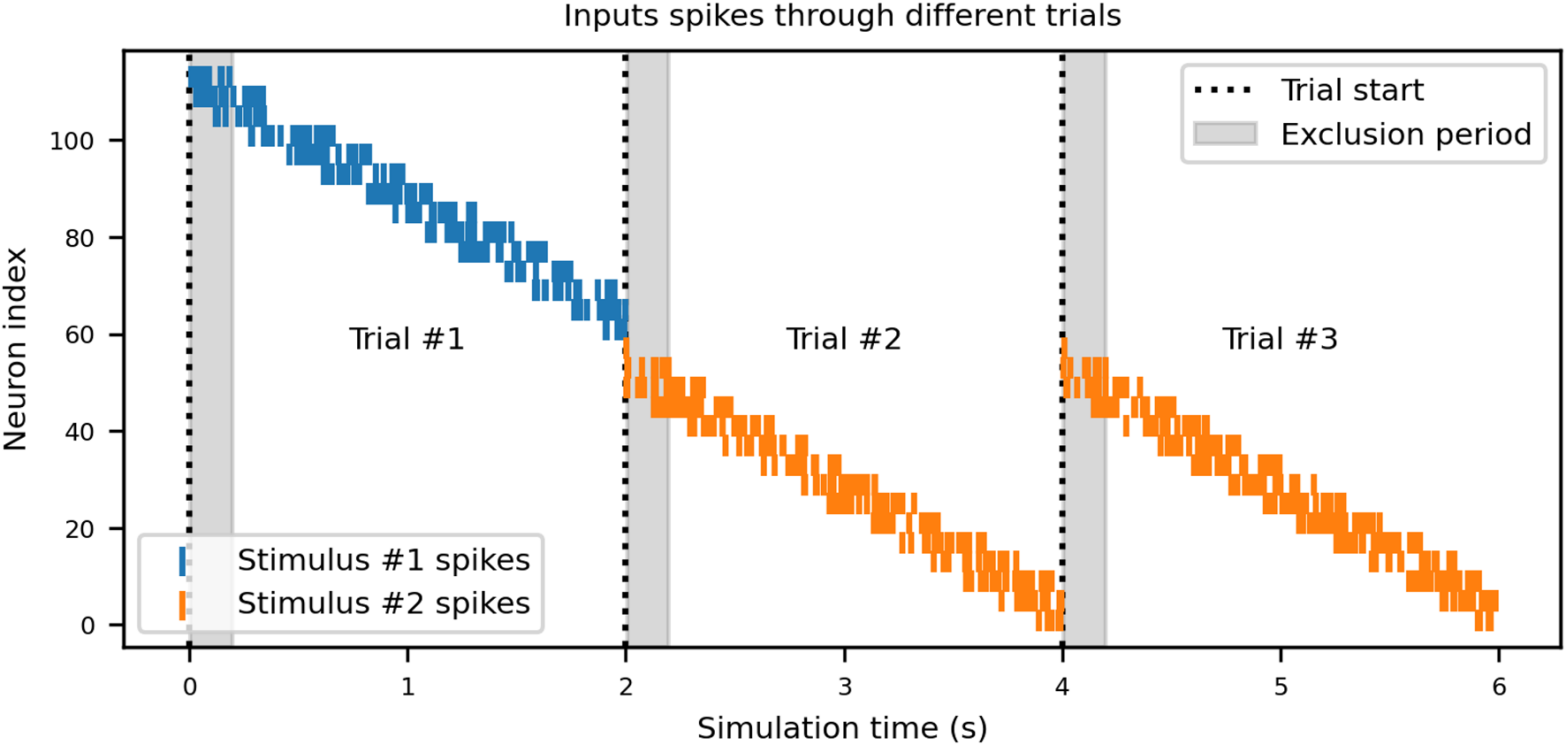
Task structure and temporal dynamics. This figure shows the temporal dynamics of the experimental task across three consecutive trials. Each trial spans 2 seconds, during which a unique stimulus elicits a distinct temporal pattern of neural activation (blue and orange lines represent spikes for Stimulus #1 and Stimulus #2, respectively). The first 200ms of each trial is designated as an *exclusion period* (gray-shaded regions), during which responses are considered premature and ignored. After this period, responses can be made during the *response period* (remaining portion of the trial). Trials are separated by boundaries at 2s and 4s (dashed vertical lines). The y-axis represents the neuron index, with each stimulus activating specific neuron populations in a sequential manner. Rewards or punishments are delivered after the response period depending on the correctness of the selected action.

Each trial includes a brief exclusion period (200 ms), during which responses are ignored, followed by a response window (see Fig. 1). Correct actions receive a delayed reward; incorrect ones are punished. These tasks probe the model’s ability to learn and adapt stimulus-action associations under increasing ambiguity and shifting contingencies.

### Model architecture

The architecture consists of parallel action channels containing direct pathway (D1) and indirect pathway (D2) striatal projection neurons (SPNs), which exhibit distinct electrophysiological properties aligned with experimental observations. D2 SPNs demonstrate enhanced excitability, supporting their role in action suppression. The output stage implements a competitive action selection circuit modulated by asymmetrical lateral inhibition, with stronger D2$\rightarrow$D1 inhibition than D1 $\rightarrow$ D2 inhibition, reflecting their complementary roles.

### Cholinergic modulation and plasticity

**Fig. 2:**
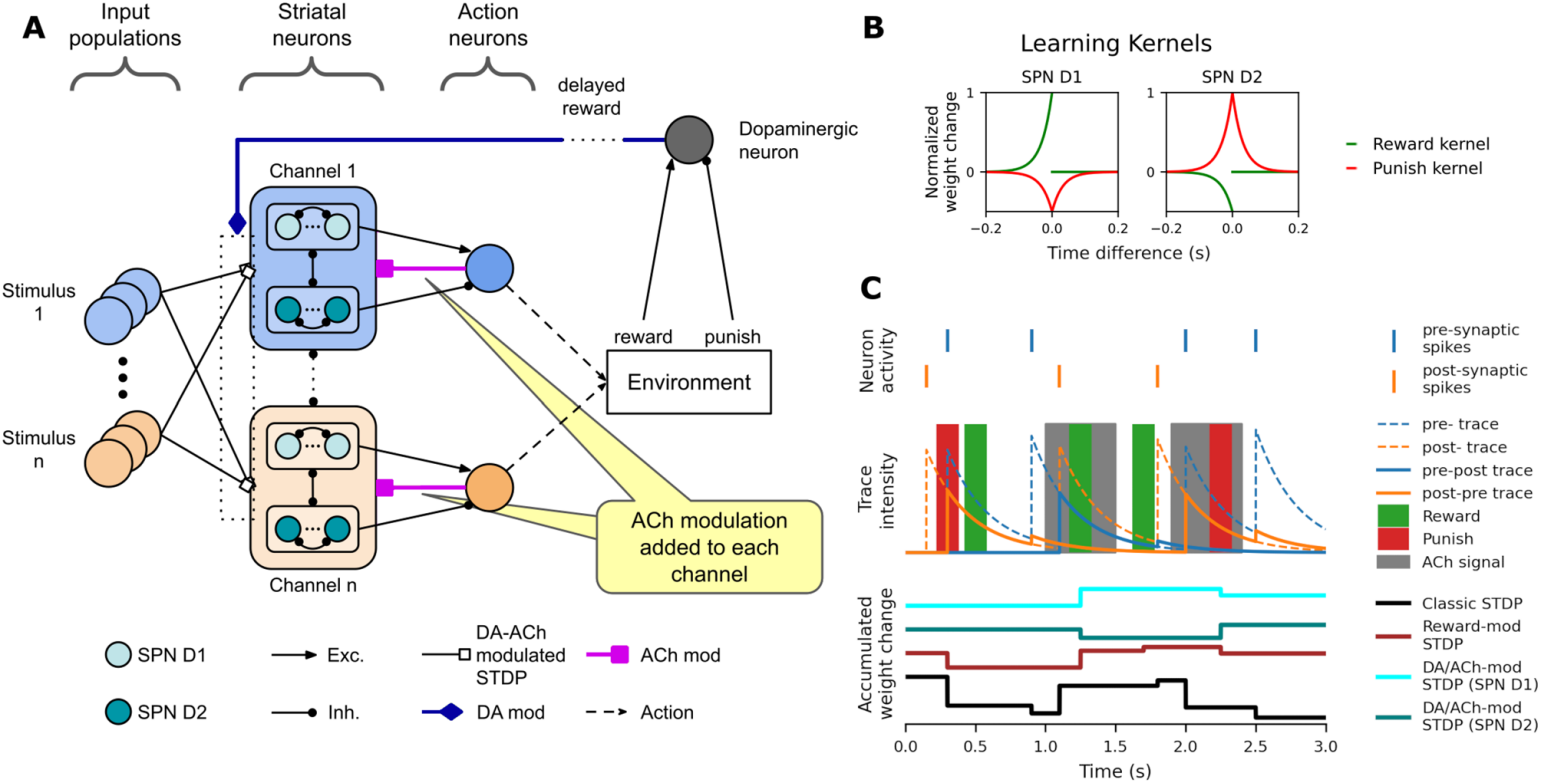
Network architecture and learning mechanisms. **(A)** Schematic of the enhanced striatal network with channel-specific cholinergic modulation. Each channel contains D1 and D2 striatal projection neurons (SPNs) receiving input from stimulus populations. D1 SPNs facilitate action selection, while D2 SPNs inhibit it, projecting to action neurons with excitatory and inhibitory effects, respectively. Action neurons trigger actions and emit local acetylcholine (ACh) signals (fuchsia) to their corresponding channels, representing a simplified CIN pause mechanism. These ACh transients gate synaptic plasticity within brief time windows. A dopaminergic population provides global reward and punishment signals (dark blue), modulating plasticity across all channels. The network includes lateral inhibition within and across channels. **(B)** Spike-timing-dependent plasticity (STDP) kernels used for synaptic updates in D1 and D2 SPNs. The x-axis indicates the time difference ($\Delta t$) between pre- and post-synaptic spikes; the y-axis shows the normalized synaptic change. Reward and punishment kernels differ across SPN types, reflecting distinct patterns of potentiation and depression. **(C)** Illustration of the plasticity mechanism dynamics. *Top:* Raster plot of pre-synaptic (blue) and post-synaptic (orange) spikes. *Middle:* Exponentially decaying traces of pre- and post-synaptic activity (dashed lines), and their interactions (solid lines) that determine STDP contributions. Gray shading indicates ACh-gated windows; green and red bars show global reward and punishment signals, respectively. *Bottom:* Accumulated synaptic weight changes under different learning rules. *Classic STDP (black):* Based solely on spike timing. *Reward-modulated STDP (brown):* Influenced by dopaminergic feedback. *DA-ACh modulated STDP (cyan for D1, teal for D2):* Plasticity is gated by local ACh signals, restricting dopamine effects to the relevant striatal channel.

The model implements channel-specific ACh gating as a hypothesis. In biological systems, pauses in cholinergic interneuron (CIN) firing–often triggered by contextual changes–produce brief, localized ACh dips that gate plasticity. Such pauses can be triggered by self-initiated voluntary movements. We assume that these self-initiated actions constitute relevant context changes that would trigger CIN pauses (see the Supplementary Note for biological rationale). In our model, we abstract this mechanism as stimulus-triggered action selection, where each action execution (always in response to a presented stimulus) triggers a corresponding cholinergic pause signal. To implement this in a computationally tractable form, each striatal “channel” includes a single action neuron that serves a dual role: triggering the action and simulating the local cholinergic pause signal (Fig. 2A).

Importantly, these action neurons do not represent CINs themselves (which are interneurons), but rather abstract the functional consequence of thalamo-striatal signaling–specifically, the CIN pauses that follow action initiation. Instead of modeling the full CM-Pf-CIN pathway^32,33^, we approximate its effect via a direct cholinergic gating signal triggered by the corresponding action neuron. Our action neurons collapse this multi-synaptic cascade into a single signal that: (1) initiates the behavioral action and (2) opens a 150ms cholinergic window in the corresponding channel. This abstraction preserves the essential temporal gating function without explicitly modeling tonic CIN firing or thalamic dynamics.

Learning in the model follows a three-factor plasticity rule combining spike timing, cholinergic gating, and dopaminergic feedback (Fig. 2B,C). Synaptic updates occur within brief cholinergic windows, triggered by action neuron spikes and validated by delayed dopaminergic signals. This mechanism abstracts the temporal gating function of CIN pauses, while omitting the detailed dynamics of their tonic firing. To prevent uncontrolled synaptic growth and ensure long-term stability, we incorporate a biologically motivated multiplicative LTD term that scales weights according to postsynaptic activity. This form of LTD approximates biologically observed synaptic scaling mechanisms^34^ and is conceptually related to theoretical models of weight normalization^35^. Full implementation details, including equations and parameter values, are provided in Supplementary Methods (see *Supplementary Table S2* for full parameter values). The biological evidence supporting these modeling choices is detailed in the Supplementary Note.

### Training protocol and evaluation

The model was evaluated across a series of tasks designed to probe association learning, structural credit assignment, and behavioral flexibility. We define *behavioral flexibility* as the ability to modify learned stimulus-action associations in response to changing environmental contingencies, measured by the speed and accuracy of adaptation following contingency reversals. All simulations used the same architecture and learning rules, with plasticity gated by dopamine and channel-specific acetylcholine signals (see Supplementary Methods for details). Trials consisted of 2-second episodes during which the model received stimulus input and was required to generate an appropriate action to receive reinforcement.

Three experimental settings were used:

- **Baseline learning:** two stimuli mapped to two action channels (2 channels total), with no distractors or reversals (10,000 episodes).
- **Structural credit assignment:** relevant stimuli presented with 0-8 distractors; 2 action channels; no reversals; 100 simulations per condition.
- **Contingency reversal tasks:** a reversal was introduced at episode 500 (single reversal) or every 2,000 episodes (repeated reversals), with 8 distractors and 2 action channels.

Full simulation conditions are summarized in *Supplementary Table* *S1*.

In each case, synaptic weights, population activity, and task performance were recorded. Accuracy curves and weight trajectories were smoothed using a Gaussian filter ($\sigma = 10$ episodes). For the single-reversal task, statistical comparisons were performed across 50 simulations using paired t-tests and Cohen’s *d* to assess the emergence, suppression, and remapping of stimulus-action associations. Full statistical methods and parameter values are provided in the Supplementary Material.

## Results

### Channel-specific cholinergic modulation enhances synaptic specificity

**Fig. 3:**
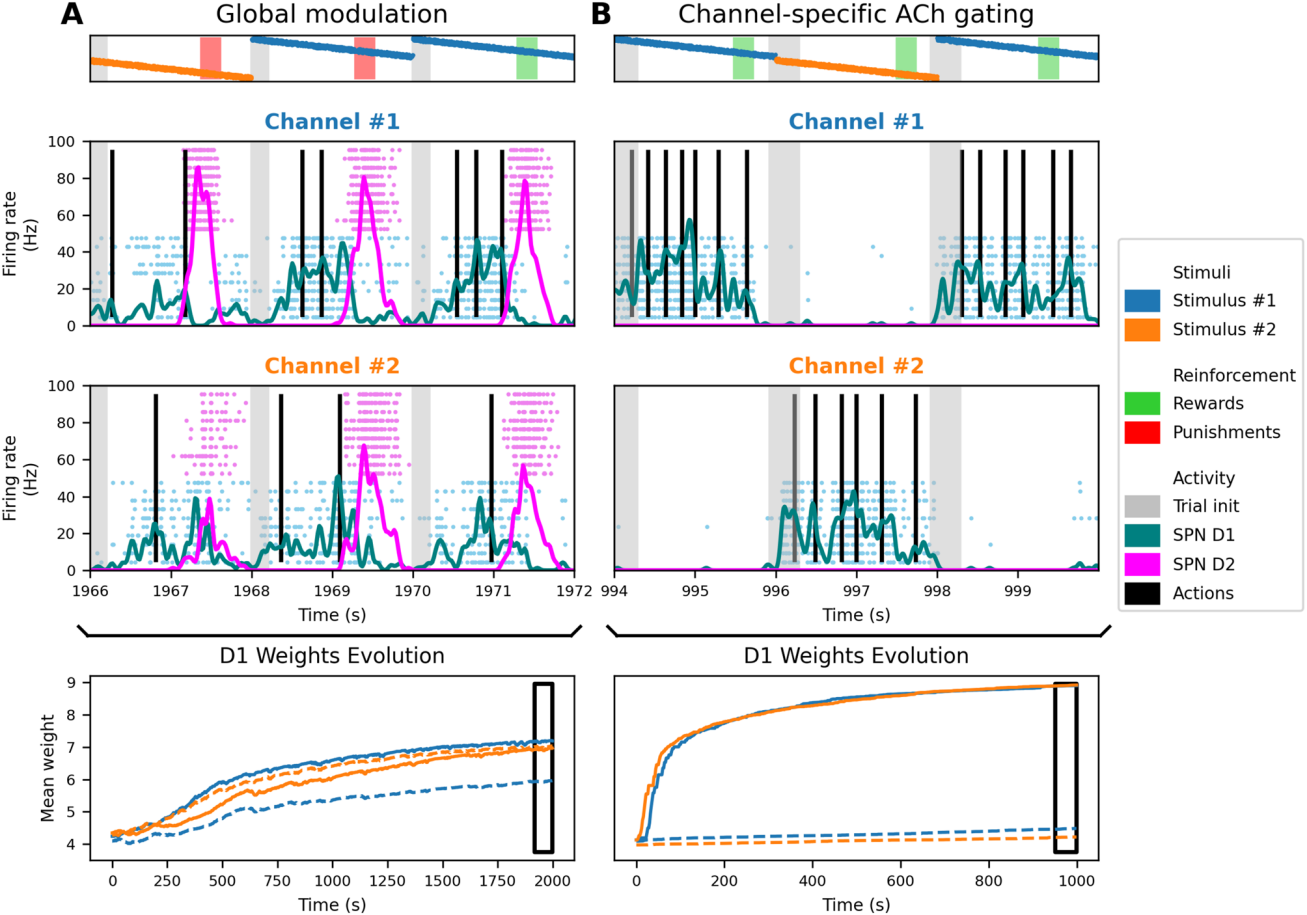
Performance comparison between global modulation and channel-specific cholinergic gating models. **(A)** Performance of the previous global modulation model in a two-choice task. *Top:* Neural activity near the end of a 2000-second simulation, showing trial starts (gray bars), stimulus presentations (Stimulus #1 and Stimulus #2), reward/punishment events (green/red shaded regions), and spiking activity of D1 (light blue) and D2 (pink) neurons in each channel. Black vertical lines indicate action triggers. Channels are color-coded to their associated stimuli (e.g., Channel #1 is blue for Stimulus #1, and Channel #2 is orange for Stimulus #2). The lack of stimulus-specific neural responses is evident in how Stimulus #1 activates D1 neurons in both channels similarly, leading to simultaneous action selection in both channels (correct in Channel #1, incorrect in Channel #2, causing punishment). This indicates that the global modulation model does not enforce channel specificity, resulting in interference between actions. In contrast, the cholinergic gating model (B) enforces channel specificity: each stimulus activates D1 neurons only in its corresponding channel, while D2 neurons remain silent, preventing incorrect action selection and unnecessary suppression. *Bottom:* Evolution of mean synaptic weights for each stimulus-channel combination (colored lines) over the course of training. Weights remain overlapping, and learning convergence is incomplete. The dark rectangle highlights the time period shown in the top panel. **(B)** Performance of the channel-specific cholinergic gating model under identical conditions. *Top:* Neural activity shows distinct stimulus-specific responses in D1 and D2 neurons. Each stimulus elicits a unique activation pattern in its associated channel, while D2 neurons remain inactive due to no suppression requirement. *Bottom:* Weight evolution demonstrates clear convergence, with strong synaptic connections forming only between each stimulus and its corresponding channel, while non-target weights remain low. This separation highlights the model’s enhanced specificity and efficiency.

Building on our previous implementation^36^, we addressed a key limitation related to stimulus-action interference. In that earlier version, global modulation during reward delivery caused all channels involved in an action to undergo synaptic strengthening–regardless of which stimulus triggered it. This non-specific plasticity led to weight increases not only for the current stimulus, but also for any stimulus previously associated with the same action, as reflected in the non-specific increases in D1 and D2 firing rates across channels (Fig. 3A). D1 and D2 populations exhibited overlapping activation for distinct stimuli, and synaptic weights converged poorly due to mutual reinforcement across channels.

To resolve this, we introduced a mechanism of channel-specific cholinergic modulation. When an action is executed, a transient cholinergic pause is triggered locally in the corresponding channel, restricting plasticity to a brief temporal window during which dopaminergic modulation is effective. This architecture enforces spatial specificity: only synapses within the activated channel are eligible for plasticity when cholinergic gating and dopamine coincide with ongoing spiking activity (Fig. 2).

This gating mechanism abstracts known properties of cholinergic interneurons (CINs), which fire tonically but can pause in response to salient events. In dorsomedial striatum, such pauses have been observed in association with self-initiated actions^13^, suggesting a potential basis for action-triggered, spatially confined cholinergic gating. Dopaminergic bursts accompanying these actions may further promote CIN desynchronization, enabling local plasticity while suppressing non-relevant pathways.

As shown in Fig. 3B, this architecture yields distinct, stimulus-specific neural responses and selective synaptic reinforcement, with minimal cross-talk between channels. Synaptic weights converge rapidly to stable configurations, reflecting independent acquisition of multiple associations. This specificity not only mitigates interference but also enables the model to scale to more complex environments, as explored in the following sections. Learning performance remained robust across different trial durations, with only minor differences in convergence speed (see Supplementary Fig. S2).

### Credit assignment under sensory ambiguity

To evaluate whether channel-specific cholinergic modulation enables the resolution of structural credit assignment, we designed a task in which multiple stimuli are presented simultaneously in each trial, but only one is behaviorally relevant and predictive of reward. The remaining stimuli act as distractors. Although the stimuli are concurrent, their association with delayed reward transforms the task into a temporal credit assignment problem as well. The agent must select a single action based on this compound input, and only the action associated with the target stimulus is reinforced. This setup therefore combines both structural and temporal credit assignment challenges, requiring the agent to identify the relevant input and link it to a delayed outcome.

**Fig. 4:**
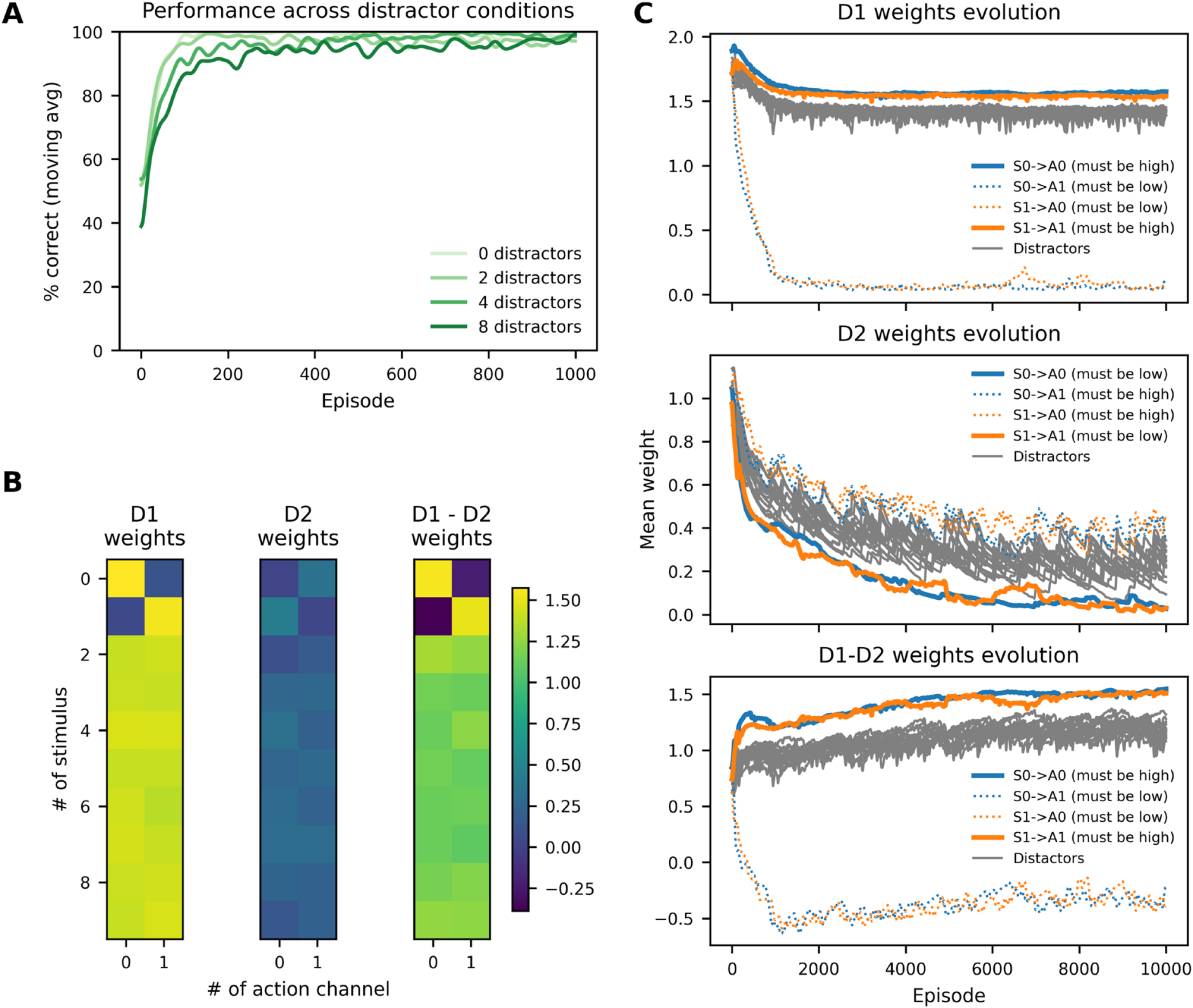
The model learns stable and selective mappings despite irrelevant stimuli. **(A)** Model performance is shown over the first 1000 episodes as the number of distractors increases from 0 to 8, with 100 seeds per condition. Curves represent the mean percentage of correct actions, smoothed with a Gaussian filter ($\sigma = 10$ episodes). Although learning slows with increasing sensory ambiguity, the network consistently acquires the correct stimulus-action mapping, even in the presence of irrelevant stimuli. **(B)** Average weight matrices at the end of the training (10000 episodes) of a single experiment. Each row corresponds to one of ten stimulus subpopulations (two relevant, eight distractors); columns represent two action channels. The D1 matrix shows strong diagonal weights, indicating selective reinforcement of relevant stimulus-action pairs. The D2 matrix shows strong anti-diagonal weights for relevant stimuli, reflecting suppression of incorrect actions. The D1-D2 matrix confirms net selectivity for correct mappings. **(C)** Temporal evolution of synaptic weights across learning of a single experiment. For correct stimulus-action pairs, D1 weights *(top)* increase and D2 *(middle)* weights remain suppressed, producing growing net selectivity (D1-D2, *bottom*). In contrast, connections from distractors show moderate or decaying weights. All synaptic weights converge to stable asymptotic values, a result of the multiplicative post-synaptic LTD term introduced in the model. This mechanism prevents unbounded potentiation and ensures long-term stability without requiring additional competitive or reward prediction error-based mechanisms.

Fig. 4A shows model performance as the number of distractor stimuli increases from 0 to 8. The network successfully learns the correct stimulus-action mapping in all conditions. While learning speed decreases with added ambiguity, final performance remains high, demonstrating the model’s ability to resolve credit assignment even under high sensory interference. Notably, this experiment omits the model variant without cholinergic gating, as previous results (see Fig. 3) already demonstrated its inability to achieve stimulus-specific learning.

To confirm that credit is assigned specifically to the relevant stimulus, we analyzed the learned synaptic weight matrices from stimulus populations to D1 and D2 neurons (Fig. 4B). The D1 matrix exhibits strong diagonal structure: each rewarded stimulus develops a dominant projection to its associated action channel. Distractor stimuli produce weaker, diffuse connections. The D2 matrix shows a complementary pattern, with off-diagonal suppression of incorrect associations. The difference matrix (D1-D2) reveals a clear sharpening of selectivity for behaviorally relevant pathways.

Finally, we confirmed that learned weights reach stable asymptotic values (Fig. 4C) due to the multiplicative post-synaptic LTD term, which prevents unbounded growth. For rewarded stimuli, D1-D2 selectivity increases over time, while distractor weights remain moderate or decay.

These results demonstrate that the model maintains selective reinforcement of behaviorally relevant inputs despite the presence of distractors, with synaptic specificity emerging robustly across simulations (Fig. 4).

### Scalability trends and comparative analysis

**Fig. 5:**
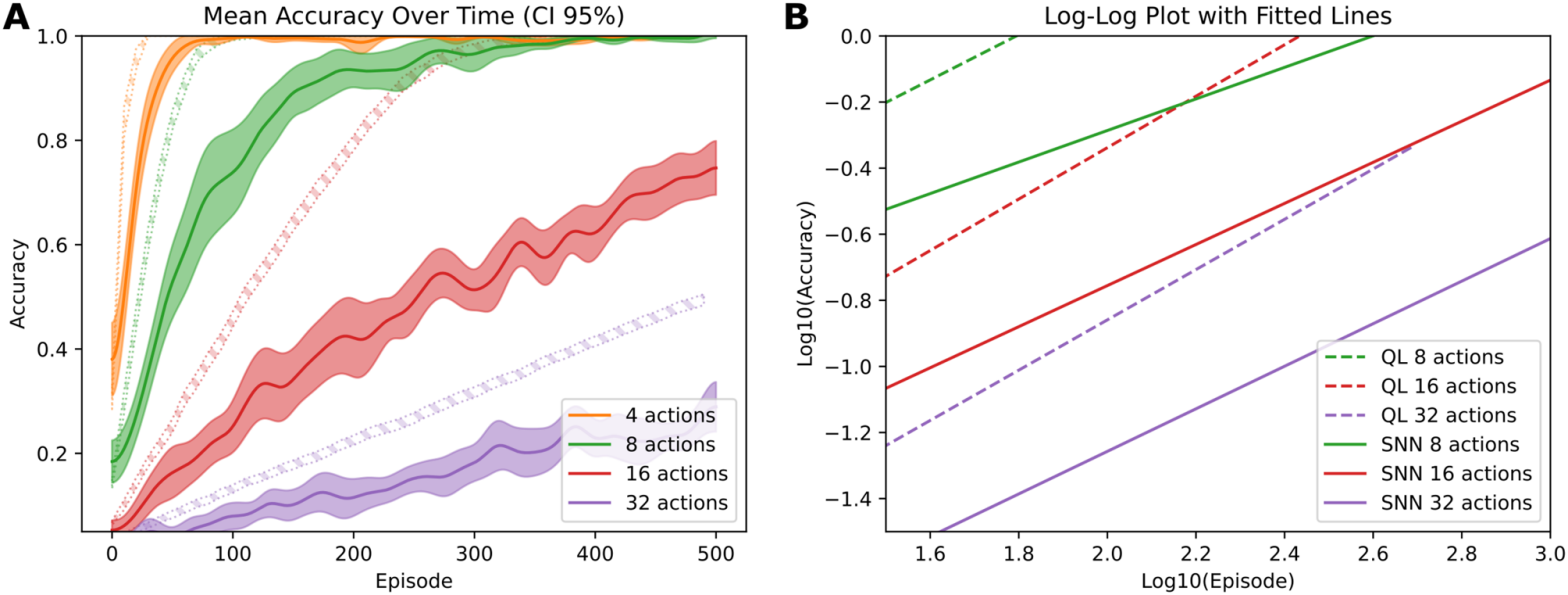
Learning performance and scaling analysis. **(A)** Mean accuracy over time for Q-Learning (QL) and spiking neural network (SNN) agents across different numbers of actions (4, 8, 16, 32), with 95% confidence intervals shown as shaded regions. Dashed lines represent QL performance, solid lines represent SNN performance. **(B)** Log-log plot analyzing early learning dynamics (accuracy < 79%) for tasks with different action spaces. Linear regressions (straight lines) on this regime reveal power-law scaling behavior, with QL agents (dashed lines) showing consistent slopes (0.707-0.791, $R^2 \ge 0.991$) across task complexity, while SNN agents (solid lines) exhibit steeper slopes (0.285-0.644, $R^2 \ge 0.870$) in more complex tasks, indicating that the relative efficiency of the SNN improves as task complexity increases–likely because the fixed computational overhead becomes less limiting.

Having established how channel-specific cholinergic modulation enables precise credit assignment, we next investigated whether this mechanism could support learning in increasingly complex action spaces. Comparing our spiking neural network (SNN) model against a standard Q-learning (QL) implementation across tasks of increasing complexity (Fig. 5), we found that both approaches were able to learn effectively across conditions, although QL consistently reached higher performance. We verified that the Q-learning baseline parameters provided consistent performance across task complexities through systematic parameter variation (see Supplementary Fig. S3). Simpler tasks (4 and 8 actions) reached near-perfect accuracy within the simulation timeframe, whereas more complex scenarios (16 and 32 actions) exhibited consistent power-law scaling in their learning curves (Fig. 5B), with sustained linear trends in log-log space throughout training. This suggests that, in principle, perfect accuracy would eventually be achieved in these harder tasks given sufficient time.

While QL consistently outperformed the SNN in terms of absolute accuracy and convergence speed, the relative performance gap diminished with increasing task size (Fig. 5A). This pattern suggests that the SNN’s fixed computational overhead–stemming from the temporal integration of spikes and indirect action competition–has a proportionally smaller impact in large action spaces, where exploration demands dominate learning efficiency.

We further analyzed early learning dynamics by focusing on the low-accuracy regime (accuracy <79%, corresponding to log$_{10}$(accuracy) <-0.1), which best reveals power-law behavior. QL maintained consistent scaling slopes across conditions (0.707-0.791, $R^2 \ge 0.991$), while the SNN exhibited steeper slopes in more complex tasks–from 0.285 ($R^2 = 0.870$) with 4 actions to 0.644 ($R^2 = 0.960$) with 32–suggesting that its relative efficiency improves as task complexity increases.

These results suggest that channel-specific cholinergic modulation enables credit assignment while maintaining competitive scaling behavior as task complexity increases. The coordinated action of dopaminergic and cholinergic signals supports learning dynamics that remain effective in increasingly challenging environments.

### Contingency reversals and synaptic adaptation

To evaluate the model’s behavioral flexibility, we tested its ability to adapt to repeated reversals in stimulus-action contingencies. In this task, stimuli initially mapped to specific actions were remapped multiple times during training, requiring the model to suppress previously rewarded associations and acquire new ones without resetting synaptic weights. This setup probes whether the learning dynamics can support both forgetting and relearning in a continuous manner.

**Fig. 6:**
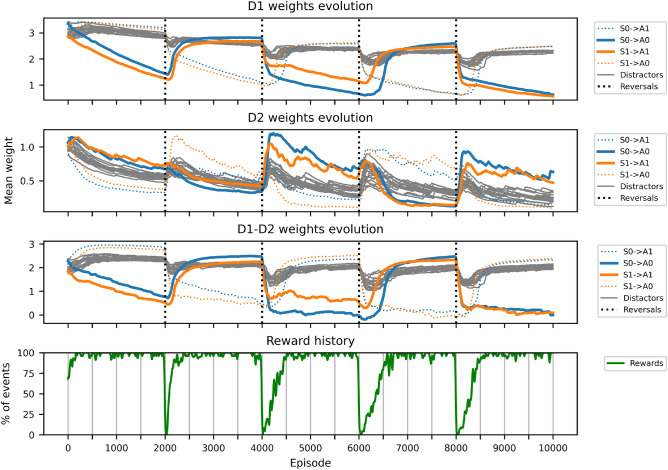
Evolution of stimulus-action associations across repeated contingency reversals. Each colored curve tracks the average weight of D1 neurons *(top)*, D2 neurons *(second row)*, and net effective weight (D1 minus D2, *(third row)*) from one stimulus population to one action channel. Initially, the two relevant stimuli (S0 and S1) are associated with actions A0 and A1, respectively, leading to selective potentiation of the corresponding connections. At four points during training (marked by black dotted vertical lines), stimulus-action contingencies are reversed. The model consistently adapts by weakening previously reinforced connections and strengthening the new correct ones. Weights associated with distractor stimuli (gray curves) remain intermediate and unspecific, demonstrating that learning is confined to behaviorally relevant input pathways. *(Bottom)* Reward history shows the percentage of rewarded trials (green line, smoothed with $\sigma = 10$ episodes), illustrating sharp performance drops immediately after each reversal followed by gradual recovery as the network adapts to new contingencies.

**Fig. 7:**
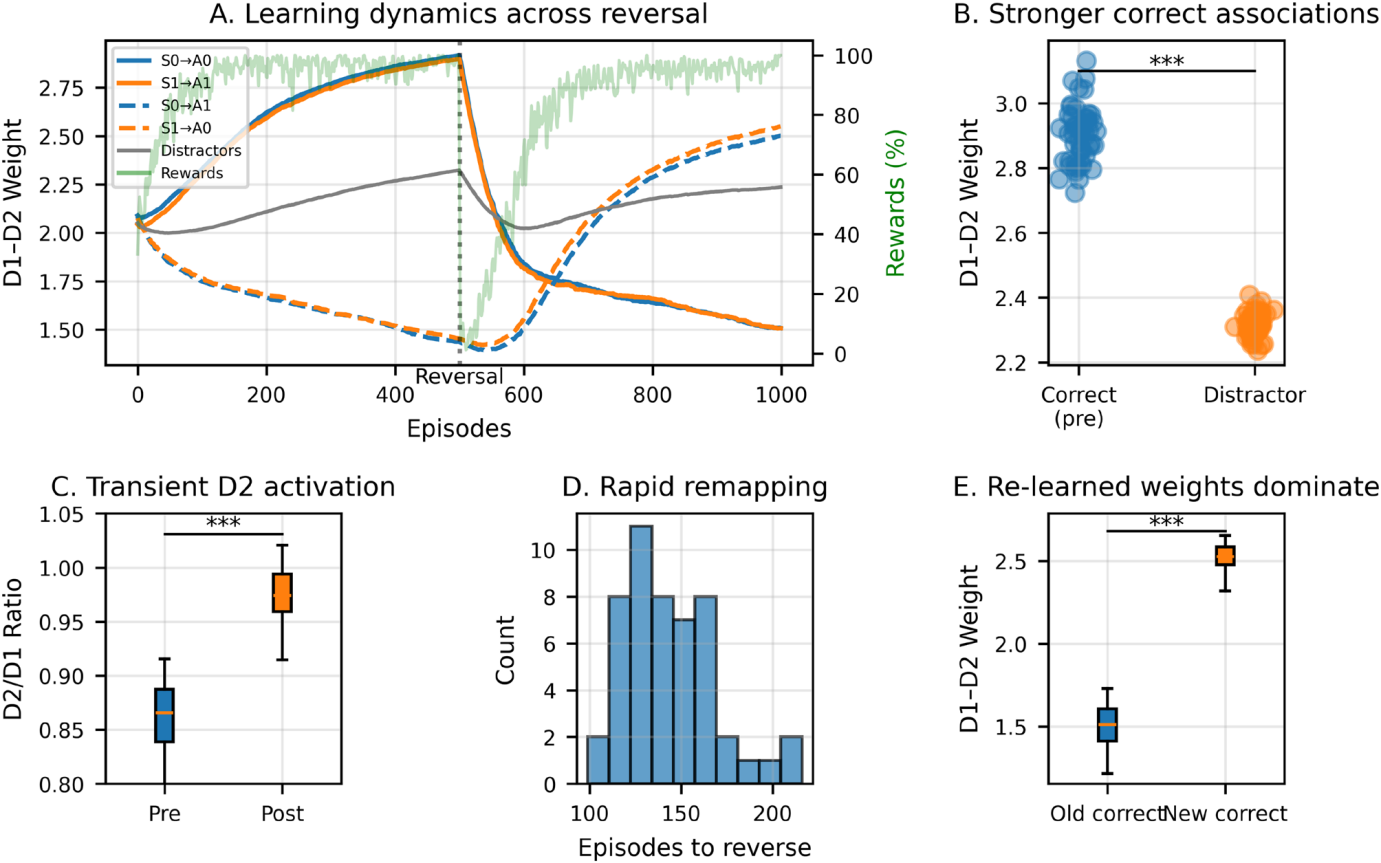
Adaptive credit assignment across contingency reversals. **(A)** Evolution of D1-D2 synaptic weights averaged across 50 simulations for relevant (S0$\rightarrow$A0, S1$\rightarrow$A1) and irrelevant (distractor) stimulus-action pairs over training. Dashed lines represent associations that become correct after the reversal at episode 500 (dotted line). The green line shows behavioral performance (percentage of rewarded trials, smoothed with $\sigma = 10$ episodes), demonstrating the sharp drop and recovery in task accuracy during adaptation. **(B)** Prior to reversal, correct stimulus-action connections developed significantly stronger weights than distractor connections (paired t-test: t(49) = 42.72, ***p*** < 0.001, Cohen’s *d* = 6.1). **(C)** The D2/D1 activity ratio increased significantly after reversal (t(49) = -33.32, ***p*** < 0.001), consistent with transient engagement of the indirect pathway to suppress obsolete responses. **(D)** The model successfully remapped stimulus-action associations within 144.1 ± 25.4 episodes following reversal. **(E)** By the end of training, newly correct connections (2.53 ± 0.08) significantly exceeded previously correct connections (1.51 ± 0.12, p < 0.001), demonstrating complete behavioral adaptation. Asterisks indicate ***p*** < 0.001.

To quantitatively assess the model’s capacity for credit assignment and behavioral adaptation, we conducted 50 independent simulations with 8 distractor stimuli and a single contingency reversal at episode 500 (see Methods and Fig. 7). Prior to reversal, correct stimulus-action connections (e.g., S0$\rightarrow$A0, S1$\rightarrow$A1) developed significantly stronger D1-D2 weights (2.90±0.09) than distractor connections (2.32±0.04; paired t-test: *t*(49)=42.7, *p*<0.001, Cohen’s *d*=6.1). Following the reversal, the D2/D1 activity ratio increased significantly (*t*(49)=-33.3, *p*<0.001), consistent with transient engagement of the indirect pathway to suppress outdated responses. The model successfully remapped the stimulus-action associations within 144.1 ± 25.4 episodes, with newly correct connections (2.53 ± 0.08) eventually exceeding previously correct ones (1.51 ± 0.12; *t*(49)=57.2, *p*<0.001). These results confirm that channel-specific cholinergic modulation enables both precise credit assignment and flexible adaptation, complementing the qualitative dynamics observed in Fig. 6.

The model includes a multiplicative postsynaptic LTD mechanism that stabilizes learning by driving weights toward equilibrium, preventing the reemergence of obsolete associations after prolonged training. This mechanism ensures long-term stability without requiring resets.

**Fig. 8:**
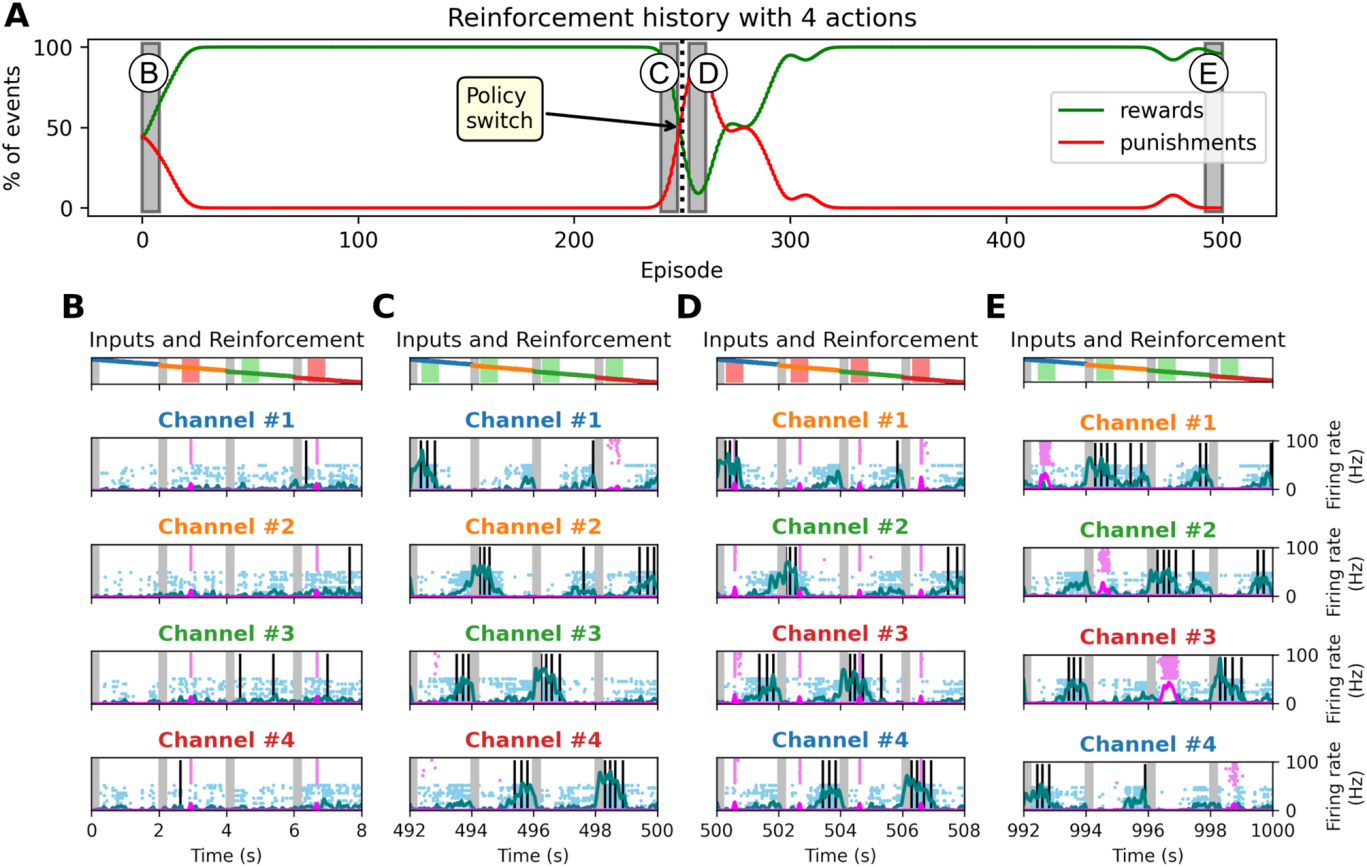
Representative striatal dynamics across a contingency reversal. This figure shows a shorter simulation (500 episodes total) with a reversal at episode 250, used to illustrate neural dynamics more clearly. **(A)** Evolution of rewards (green) and punishments (red) over 500 episodes, illustrating rapid learning and adaptation after a contingency reversal at episode 250. Channel assignments for each stimulus are indicated by label colors, which shift after the contingency reversal to reflect the change in the rewarded channel. Grey rectangles highlight time windows expanded in panels B-E. **(B-E)** Detailed neural dynamics during key learning phases. Each panel shows stimulus presentation (top), reward/punishment outcomes (green/red dots), D1 (blue) and D2 (pink) SPN activity across action channels, and triggered actions (black lines). **(B)** Early learning: exploratory D1 activity across channels with frequent incorrect responses. This initial widespread D1 activation across channels reflects exploratory behavior, as the network samples multiple actions before converging on the correct stimulus-action pair. **(C)** Late pre-reversal phase: D1 activity is stimulus-specific and dominant; D2 suppressed. **(D)** Immediately after reversal: persistent D1 activity from previous associations coexists with emerging D2 activation to suppress outdated responses. **(E)** Post-adaptation: D1 SPNs remap to newly rewarded channels; D2 activity reflects residual suppression of old associations. This temporal sequence highlights the interplay between reinforcement and suppression mechanisms during behavioral adaptation.

We examined how this behavioral adaptation unfolds at the neural level by analyzing striatal activity patterns during contingency reversals. Neural activity patterns during reversal events reveal how behavioral switching unfolds over time (Fig. 8). For visualization purposes, this simulation used a shorter training duration (500 episodes) with a reversal at episode 250. During early learning, D1 activity is diffuse across channels, reflecting exploration. Once a stimulus-action mapping is acquired, D1 responses become focused and dominant. After a reversal, D2 neurons transiently activate to inhibit the outdated pathway, while D1 activity remaps to the newly rewarded channel. This dynamic reflects coordinated engagement of reinforcement and suppression mechanisms, mediated by the DA-ACh-SPN interplay.

Together, these results demonstrate that localized cholinergic gating not only enhances stimulus specificity during acquisition but also supports flexible reconfiguration of learned associations in response to environmental change. The synaptic weight analyses reveal how D1 pathways maintain stimulus-specific potentiation while D2 pathways transiently activate to suppress obsolete associations–a division of labor that emerges naturally from the interplay between channel-specific acetylcholine and global dopamine signals. This coordinated plasticity enables the network to adapt rapidly to contingency reversals without catastrophic forgetting of the overall task structure, illustrating how biologically inspired mechanisms can support both specificity and flexibility in reinforcement learning.

## Discussion

### Biological mechanisms of credit assignment

These results demonstrate that channel-specific ACh modulation provides a computational solution to the structural credit assignment problem. By restricting plasticity to the striatal channel responsible for action execution, the mechanism prevents interference from competing stimuli and enables selective reinforcement of behaviorally relevant pathways. This gating relies on a brief, channel-specific cholinergic pause that acts in combination with a global dopaminergic signal and local neural activity, forming a three-factor learning rule that aligns with known biological constraints^9,37^.

The improvement over our previous model without ACh modulation^36^ highlights the computational value of spatially restricted neuromodulation: by confining plasticity to the behaviorally active channel, the system avoids interference and enables scalable reinforcement learning^15,26,27^.

This mechanism also reflects known biological asymmetries in striatal function: D1 SPNs in the direct pathway consolidate stimulus-action associations, while D2 SPNs in the indirect pathway transiently suppress outdated responses^24,25^.

Together, these findings provide a computational account of how localized cholinergic modulation, combined with dopaminergic reward signals, can solve credit assignment in distributed corticostriatal circuits. See Supplementary Note for supporting biological evidence.

### Trade-offs and limitations

While the model replicates key features of striatal plasticity and credit assignment, it also highlights important trade-offs that arise when enforcing long-term stability. The multiplicative postsynaptic LTD mechanism ensures stability by preventing unbounded weight growth. However, this also reveals a fundamental trade-off: previously learned associations are erased rather than merely suppressed after contingency reversals. As a result, the model lacks spontaneous recovery, consistent with behavioral findings that support erasure-based updating in the striatum^38^.

Our operational definition of behavioral flexibility–the ability to adapt stimulus-action associations following contingency reversals–captures only one aspect of the broader flexibility observed in biological systems. Natural behavioral flexibility encompasses additional capabilities including set-shifting between different task rules, switching between multiple behavioral contexts, and adapting to partial or probabilistic changes in reward contingencies. These more complex forms of flexibility likely involve additional neural circuits beyond the striatum, including prefrontal cortical regions, and represent important directions for future modeling efforts.

This trade-off is consistent with recent in vivo evidence in rodents showing that, after contingency reversals, prior stimulus-action associations do not spontaneously re-emerge. Reinhold et al.^38^ found that the dorsomedial striatum (DMS) is required for acquiring new associations after a reversal, but not for retrieving previously learned responses. Notably, prior associations were not recovered even after inactivating the DMS, suggesting that original memories had been functionally erased rather than simply suppressed. This supports the idea that the striatum plays an essential role in updating associations during reversal learning, and that striatal plasticity mechanisms may prioritize adaptability over long-term retention.

Although this trade-off reflects known biological dynamics, our model likely amplifies it due to the simplified reward structure, where dopamine signals remain constant even after near-perfect performance is achieved–unlike biological RPE signals that diminish for predicted outcomes^39^. Incorporating realistic RPE dynamics (see Supplementary Methods) could allow synaptic weights to stabilize without full erasure, potentially explaining phenomena like savings in reacquisition.

Beyond these internal learning dynamics, the model makes several simplifying assumptions. We do not simulate attentional processes that would restrict the number of stimuli that can be simultaneously processed, nor do we incorporate working memory limitations or other constraints on synaptic maintenance. These omissions likely contribute to our model’s ability to handle up to 32 stimulus-action pairs–a capacity that exceeds what would be expected in many biological contexts, where attentional focus and working memory limitations constrain the number of simultaneously maintained associations to approximately 3-4 items^40,41^. Future work should explore how these additional biological constraints interact with cholinergic modulation to shape learning in complex environments, potentially revealing optimal trade-offs between flexibility and capacity shaped by evolutionary pressures.

Related to these capacity limits, the model’s learning speed ($\tilde{1}$00 episodes for simple associations) may appear slow but reflects our conservative parameter choice to ensure stable dynamics and clear visualization of pathway-specific contributions. While tenfold increases in learning rate yield  5-fold faster learning (Supplementary Fig. S1), they also introduce noise that obscures the mechanistic insights central to this study.

Our scalability analysis suggests that the fixed overhead introduced by biologically inspired constraints–such as temporal spike integration and indirect action competition–becomes less limiting as task complexity increases. In large action spaces, where exploration demands dominate, the efficiency of channel-specific credit assignment approaches that of optimal tabular methods like Q-learning.

These simplifications, while limiting biological realism, allow us to isolate and examine the computational advantages of channel-specific gating. Our results reveal an important principle: by restricting plasticity to causally relevant circuits, biological systems can maintain multiple stimulus-action mappings without catastrophic interference. This suggests that CIN-mediated gating may serve to support parallel learning in high-dimensional environments by restricting plasticity to causally relevant circuits.

### Future work and testable predictions

One promising direction is to investigate how dopaminergic tone may influence CIN synchrony. In our model, high dopamine levels following successful actions are abstracted as enabling spatially localized CIN pauses. This is consistent with experimental evidence suggesting that dopamine can reduce coupling between cholinergic interneurons–via D2 receptor modulation of THIN (tyrosine hydroxylase-expressing interneuron)-mediated lateral inhibition–thereby promoting desynchronization and spatial confinement of pauses^18^. A testable prediction is that CIN pauses should become more localized during reward-driven behaviors compared to neutral or exploratory conditions, particularly in dorsomedial striatum^13^.

Another open question is how to balance synaptic stability with memory retention. As shown in our results, multiplicative LTD enforces long-term stability but eliminates prior associations during contingency reversals. In contrast, a biologically grounded RPE signal may allow plasticity to decline once an association is learned, preserving prior weights unless a new error is detected. Future work should explore models combining local cholinergic gating with RPE-modulated plasticity to support both adaptability and memory conservation. Such models may capture more accurately the extinction-reacquisition dynamics observed in behavioral experiments^42^.

From a systems perspective, it would be valuable to examine how channel-specific plasticity scales under more naturalistic constraints. These include partial observability, delayed rewards, action hierarchies, and neuromodulatory noise. Integrating this model into hierarchical architectures or actor-critic frameworks may reveal how localized credit assignment interacts with cortical-basal ganglia loops during complex decision-making. For modeling behavioral variability observed in animals and humans more accurately, future work should explore softmax decision functions that incorporate relative value differences, rather than the $\varepsilon$-greedy policy used here for computational simplicity.

Finally, the model generates concrete experimental predictions. For example, in tasks with multiple stimuli and a single rewarded action, cholinergic pauses should occur selectively in the striatal region representing the executed action, and plasticity should be restricted to those synapses. Likewise, in reversal learning tasks, D2 pathway activity should transiently increase during suppression of outdated responses, followed by D1 re-potentiation of newly rewarded associations. These patterns can be tested using simultaneous imaging or electrophysiological recordings of D1, D2, and CIN populations in behaving animals.

Taken together, these directions outline how the present model serves not only as a computational demonstration of channel-specific plasticity, but also as a framework for guiding and interpreting future experimental work.

## Supplementary Information

Supplementary Information.

## Data Availability

All data generated and analyzed in this study can be reproduced in full using the code and configuration files provided in the public GitHub repository: https://github.com/EduardoRosLab/STR_DA_ACh. To ensure reproducibility and environment consistency, a Docker container is also provided, including all necessary dependencies and instructions to reproduce the figures and results presented in this article.
