# Supplementary material for: Cholinergic modulation enables scalable action selection learning in a computational model of the striatum
Source: Sci Rep. 2025 Oct 7;15:34902. doi: 10.1038/s41598-025-18776-3 (PMC12504730; doi:10.1038/s41598-025-18776-3)
Supplement: Supplementary file 1 — Supplementary Information. [file 41598_2025_18776_MOESM1_ESM.pdf]

Supplementary Materials: Cholinergic Modulation Enables Scalable Action Selection Learning in a Computational Model of the Striatum

Álvaro González-Redondo[1,*], Jesús A. Garrido[1], Jeanette Hellgren Kotaleski[2,3], Sten Grillner[2], and Eduardo Ros[1]

[1]Information and Communications Technology Research Center of the University of Granada (CITIC-UGR). Department of Computer Engineering, Automation and Robotics. University of Granada. Granada, Spain
[2]Department of Neuroscience, Karolinska Institutet, Stockholm, Sweden
[3]Science for Life Laboratory, Department of Computer Science, KTH Royal Institute of Technology, Stockholm, Sweden
[*]Corresponding author: alvarogr@ugr.es

ABSTRACT

This document provides supplementary materials for the article titled "Cholinergic Modulation Enables Scalable Action Selection Learning in a Computational Model of the Striatum". It includes additional analyses, extended results, detailed model specifications, and reproducibility information.

Supplementary Note - Biological Rationale for Channel-Specific Cholinergic Modulation

The modeling approach presented here prioritizes functional abstraction over anatomical detail. Rather than simulating individual cholinergic interneurons (CINs), thalamic nuclei, or their explicit connectivity, we approximate their joint effect on plasticity by modeling channel-specific gating signals triggered by action execution. Here, we use the term *channel* to refer to a functionally segregated set of striatal neurons associated with a particular action, not to a discrete anatomical structure. This abstraction is commonly used in basal ganglia models to represent parallel action-selection pathways[1,2]. While evidence supports topographic organization and domain-specific connectivity in corticostriatal circuits, the notion of completely segregated channels remains a simplifying assumption that allows us to study credit assignment mechanisms while maintaining computational tractability. This design choice is grounded in converging evidence from multiple biological mechanisms::

Spatial specificity of cholinergic pauses. Early studies suggested that CIN pauses were globally synchronized across the striatum[3], but more recent findings demonstrate substantial spatial and functional heterogeneity. CIN responses vary depending on striatal subregion, behavioral context, and task demands[4–6]. In particular, dorsolateral CINs exhibit burst–pause–rebound sequences in response to salient external cues, dorsomedial CINs display pause–rebound patterns aligned with self-initiated movements, and ventral CINs often show no pause but instead increase firing proportionally to reward prediction errors[6,7]. Further supporting this view, recent evidence shows that CIN pause expression in the dorsolateral striatum depends both on the strength of excitatory thalamic input and on local dopaminergic modulation[8]. This indicates that CIN pauses are not uniformly generated, but rather reflect circuit-specific integration of afferent and neuromodulatory signals. Complementing this, thalamic input from the centromedian and parafascicular (CM–Pf) nuclei conveys associability signals to CINs in response to salient stimuli and contextual changes[9,10], further supporting the idea that cholinergic modulation is both spatially localized and behaviorally contingent. Together, these findings provide a plausible biological substrate for channel-specific plasticity gating.

Dopamine–CIN–THIN interaction. High dopamine levels, typically following successful actions, can promote localized CIN desynchronization. This effect is mediated by the CIN–THIN–CIN microcircuit, where THINs (tyrosine hydroxylase-expressing interneurons) are a specialized class of striatal GABAergic interneurons that mediate lateral inhibition between CINs[11]. When dopamine acts on presynaptic D2 receptors located on CIN terminals, it reduces cholinergic transmission to THINs, thereby weakening THIN-mediated lateral inhibition among CINs. This disinhibition limits the spatial extent of CIN pauses, creating more localized cholinergic modulation. CIN activity is also temporally aligned with dopaminergic transients related to movement initiation[6,12], suggesting a mechanism by which dopaminergic and cholinergic signals jointly define narrow spatiotemporal windows of plasticity eligibility.

Spatiotemporal gating of plasticity. Striatal plasticity requires the coincidence of presynaptic activity, postsynaptic depolarization, and phasic dopamine within a critical time window of 300 ms[13]. While dopamine provides a global reward signal, spatially and temporally restricted cholinergic pauses may serve as a local gating mechanism. This interaction implements

a biologically grounded three-factor learning rule that enforces synaptic specificity. The convergence of these signals aligns with experimental findings on corticostriatal plasticity[14] and theoretical proposals that global and population-specific feedback can jointly improve credit assignment efficiency[15].

Functional differentiation of D1 and D2 pathways. In addition to plasticity gating, efficient learning requires complementary functions in striatal output pathways. D1-expressing spiny projection neurons (SPNs) in the direct pathway reinforce selected actions, while D2 SPNs in the indirect pathway contribute to suppressing outdated or incorrect responses[16,17]. This asymmetry is incorporated in the model design, consistent with observations that D1 neurons develop strong, stimulus-specific associations aligned with rewarded behaviors, while D2 neurons are progressively recruited during contingency reversals to inhibit previously learned but obsolete responses. This division of labor supports adaptability and mirrors experimental observations of pathway-specific plasticity dynamics.

While the model does not simulate individual cholinergic interneurons (CINs) or thalamic projections explicitly, it abstracts their functional role through action-triggered gating signals confined to the corresponding striatal channel. This simplification captures the essential computational consequence of the CM–Pf–CIN axis and dopamine-modulated CIN desynchronization: the emergence of spatially and temporally precise windows for synaptic plasticity. By restricting plasticity eligibility to the channel responsible for action execution, the model prevents interference from irrelevant pathways and supports selective reinforcement. This mechanism enables precise assignment of credit in environments with competing stimuli and actions, offering a biologically grounded and computationally scalable solution to the structural credit assignment problem.

Supplementary Results

Learning Rate Selection

We evaluated the model's performance across different learning rates. While rates up to 1e-2 produced stable learning, we selected 1e-3 for all main experiments to optimize the trade-off between convergence speed and dynamic clarity (Figure S1).

Supplementary Methods

Task Design and Parameter Justification

Trial Structure and Stimulus Encoding

We implemented three task types to evaluate learning specificity, credit assignment, and behavioral flexibility. In all tasks, each stimulus is associated with a temporally structured pattern of activation across a population of 60 input neurons. Specifically, each stimulus drives a moving window of activity that advances one neuron every 39 ms, starting from neurons 1–10 and progressing to 51–60. This produces a stimulus-specific sequential activation pattern lasting 2000 ms per trial. Input neurons fire at rates up to 50 Hz during their active window.

During the first 200 ms of each trial (10% of the trial duration), an *exclusion period* is enforced, during which any action is ignored and receives no feedback. This models the delay between stimulus onset and action readiness. After this period, the network may trigger one of several possible actions. If the correct action is selected (i.e., the one associated with the presented stimulus), a reward of +1 is delivered after a short delay. Incorrect responses are punished with –1. Inaction within the full trial window results in no outcome.

In the *credit assignment task*, multiple stimuli (1 target and 0–8 distractors) are presented simultaneously. Only the action associated with the target stimulus is rewarded, requiring the model to resolve ambiguity and assign credit correctly. In the *reversal task*, stimulus–action mappings are permuted periodically (every 2000 trials). For example, the correct action for stimulus S_i becomes $A_{(i+1) \bmod N}$, where N is the number of stimulus–action pairs. This tests the model's ability to suppress outdated associations and learn new ones without resetting synaptic weights.

Trial Duration Robustness

To assess the temporal robustness of the learning mechanism, we evaluated model performance under varying trial durations: 1, 2, and 4 seconds. Each condition was run using 5 distinct random seeds. Figure S2 shows the mean accuracy over episodes for each duration.

All conditions yielded successful learning, with final performance levels converging to similar asymptotes. Trials of 1 second produced marginally slower convergence, likely due to reduced time for stimulus processing and evidence accumulation. In contrast, 2- and 4-second trials exhibited overlapping learning trajectories and indistinguishable final accuracy.

These results confirm that the model's credit assignment mechanism is not critically dependent on a specific trial timescale. The system tolerates a broad range of temporal conditions, provided that the stimulus–action mapping unfolds within the effective window of cholinergic modulation (150 ms). Extremely short trials may begin to challenge this requirement, but within the tested range, the mechanism remains robust.

Biological Motivation and Parameter Selection

The 2000 ms trial duration ensures sufficient temporal structure in the input for sequence discrimination. The exclusion period (200 ms) mimics an early processing window in which actions are either physically impossible or unscored. Sequential input patterns allow the model to rely on both spatial and temporal cues when mapping stimuli to actions.

The reward delay (300 ms) reflects typical feedback latencies in behavioral paradigms. The use of a punishment signal (–1) upon incorrect responses is intended to engage D2-mediated behavioral depression, consistent with the model's plasticity mechanisms. The absence of feedback for no response avoids artificially reinforcing inaction.

Parameter values for learning rates, synaptic time constants, and ACh/DA gating windows were selected to fall within plausible biological ranges, based on previous work on corticostriatal learning and STDP rules. Full parameter values and equations are provided in Table S2.

Experimental Conditions

General Training Procedure

All simulations used the core architecture detailed in the Model Architecture section. Plasticity was driven by biologically inspired gating mechanisms, see Learning Mechanisms for full details.

Each simulation lasted between 2,000 and 10,000 episodes depending on the task. One episode spanned 2 seconds of simulated time. Networks were initialized with random synaptic weights and run using different random seeds. Each condition was repeated either 50 or 100 times. Spike trains, synaptic weights, and performance metrics were recorded throughout. Unless otherwise specified, learning rates and delays were fixed across tasks. Evaluation metrics included accuracy, synaptic weight trajectories, and neural activity patterns. Gaussian smoothing ($\sigma = 10$ episodes) was applied to curves where appropriate.

Baseline Association Learning

This condition tested whether the model could form correct stimulus–action mappings in the absence of ambiguity or reversals. Two stimuli (S0, S1) were associated with two action channels (A0, A1). The model was trained for 10,000 episodes with no distractors or contingency changes. This served as a control baseline and was used to tune the plasticity parameters to ensure robust convergence.

Structural Credit Assignment under Sensory Ambiguity

To evaluate credit assignment under ambiguous input conditions, we designed a task where each trial presented one of the two relevant stimuli (S0, S1) alongside 0, 2, 4, or 8 distractor stimuli. Distractors were randomly selected from a fixed stimulus pool. Relevant stimuli were randomly assigned to A0 or A1 per simulation.

Each episode lasted 2 seconds and required the model to emit the correct action shortly after stimulus onset. Correct responses were reinforced via phasic DA release. Performance was computed as the percentage of trials where the selected action matched the assigned one.

We ran 100 independent simulations per ambiguity condition. Episode-wise accuracy was averaged and smoothed. Synaptic weights from each stimulus to D1 and D2 populations were recorded throughout. Final weight matrices were visualized after 10,000 episodes; full temporal trajectories were also analyzed.

Single Reversal Learning Task

This experiment assessed the model's capacity for behavioral flexibility following an unexpected change in stimulus–action mappings. Two relevant stimuli (S0, S1) were presented alongside 8 distractors. Initially, each relevant stimulus was assigned to one of the two action channels. At episode 500, the assignments were reversed (e.g., S0→A1 instead of A0).

Each of the 50 simulations tracked the evolution of D1–D2 synaptic weights over time. The reversal forced the model to suppress previously rewarded associations and learn new ones. Adaptation latency was defined as the number of episodes after reversal required to recover the pre-reversal peak performance level.

Statistical analysis. We computed D1–D2 effective weights for each stimulus–action pair. Paired t-tests were used to assess: (1) the difference in synaptic strength between relevant and distractor stimuli before reversal; (2) the change in the D2/D1 activity ratio immediately after reversal; and (3) the final strength of newly correct versus previously correct connections. Cohen's d was used to report effect sizes. All reported values are mean±SEM.

Repeated Contingency Reversals

To test whether the model could adapt continuously over time, we ran simulations with repeated reversals every 2,000 episodes. The relevant stimuli (S0, S1) were reassigned to the opposite action channel at each reversal point (episodes 2000, 4000, 6000, and 8000). This created alternating contingencies across the 10,000-episode training.

We measured how D1–D2 weights tracked the changing contingencies and whether the model could consistently suppress obsolete associations. Only two relevant stimuli and 8 distractors were used. Weights were averaged across 100 simulations

and smoothed over time. These results were used to support the claim that the model supports long-term, flexible learning without synaptic resets.

Overview of Experimental Settings

A summary of the simulated conditions are provided in Table S1.

Table S1. Summary of simulation conditions.

Experiment	Reversal Episodes	Distractors	Simulations	Purpose
Baseline learning	None	0	50	Control and parameter tuning
Credit assignment	None	0–8	100	Ambiguity and cue competition
Single reversal	500	8	50	Adaptation dynamics
Repeated reversals	2000, 4000, ...	8	100	Long-term flexibility

Model Architecture

Network Organization

The model implements a biologically-inspired striatal microcircuit that processes convergent inputs from cortical, thalamic, and hippocampal projections, building upon our previous work[18]. While the previous model relied on an oscillatory drive to perform current-to-phase conversion, the current implementation eschews this mechanism. Instead, we employ a more explicit input representation that directly encodes temporal patterns, making the phase-locking mechanism unnecessary.

The network organizes into parallel action channels, with configurations tested ranging from 2 to 32 channels, each representing a potential behavioral choice. Within each channel, we implement 10 D1-expressing SPNs constituting the direct pathway and 10 D2-expressing SPNs constituting the indirect pathway, along with an output layer containing simplified action neurons that represent downstream basal ganglia targets. All channels share a common input layer composed of stimulus subpopulations of 60 neurons per stimulus, encoding both sensory information and temporal context.

For computational efficiency while maintaining biological relevance, we implemented several strategic simplifications. The output stage uses a single layer of action neurons rather than modeling the full basal ganglia-thalamic loop. Similarly, D1 SPN projections are implemented as excitatory rather than inhibitory connections, preserving the functional logic of the direct pathway while reducing circuit complexity.

Neuron Models and Dynamics

We modeled SPNs using the Adaptive Exponential Integrate-and-Fire (AdEx) model, with parameters matched to experimental observations and simulations ([18,19]). The distinct electrophysiological properties of D1 and D2 SPNs reflect their specialized roles in the direct and indirect pathways. D2 SPNs exhibit a more depolarized resting potential (-84.6 mV compared to -96.2 mV in D1 SPNs) and lower membrane capacitance (102.3 pF versus 123.5 pF), making them more readily excitable under equivalent input conditions. This enhanced excitability of D2 SPNs supports their role in suppressing previously reinforced actions, especially under changing reward contingencies. The output stage implements simpler dynamics through Leaky Integrate-and-Fire (LIF) neurons, utilizing mutual inhibition to achieve winner-take-all behavior in action selection.

Inhibitory Circuits

The model incorporates three complementary inhibitory mechanisms that shape network dynamics. Fast-spiking interneurons are modeled as Poisson generators providing global inhibition at 500 Hz to all SPNs. While individual FSIs have local axonal arbors (250 μm radius;[20]) without preference for D1 or D2 populations[21], they are extensively interconnected through electrical gap junctions ([22]), forming synchronized networks that coordinate inhibition across large striatal territories. This electrical coupling justifies our abstraction of FSIs as a global signal rather than modeling individual neurons, preserving their biological function as a widespread homeostatic control system. We are also following Vogels et al.[23] in using FSI-mediated inhibition for homeostatic plasticity. Within channels, we implemented asymmetric lateral inhibition between SPN populations based on experimental observations from Burke et al.[24]: D2→D1 inhibition employs a strong weight of 10.0, while D1→D2 inhibition uses a moderate weight of 5.0, reflecting the specialized roles of direct and indirect pathways in action selection. Between channels, SPNs of the same type provide mutual inhibition with a weight of 3.0, supporting action selection by implementing competition between alternatives while maintaining appropriate response specificity.

Network Initialization

Synaptic weights were initialized through empirical testing to provide stable baseline activity while allowing sufficient dynamic range for learning. The direct (D1) pathway uses weights drawn from a uniform random distribution between 0.2 and 8.0, while

Category	Parameter	Value	Units
**Trial Parameters**			
	Trial duration	2000	ms
	Exclusion period	200	ms
	Feedback delay (reward/punishment)	300	ms
	Contingency reversal interval	2000	trials
**Stimulus Encoding**			
	Input neurons per stimulus	60	neurons
	Active neurons per time step	10	neurons
	Neuron firing rate during activation	up to 50	Hz
	Step size of moving window	1	neuron / 39 ms
**Network Structure**			
	D1 SPNs per channel	10	neurons
	D2 SPNs per channel	10	neurons
	Action neurons per channel	1	neuron
	Number of channels tested	2–32	-
**Synaptic Parameters**			
	Synaptic delay range	1–66.7	ms
	D2 $\rightarrow$ D1 inhibition	10.0	arbitrary units
	D1 $\rightarrow$ D2 inhibition	5.0	arbitrary units
	Between-channel inhibition	3.0	arbitrary units
	D1 initial weight range	0.2–8.0	arbitrary units
	D2 initial weight range	0.05–2.0	arbitrary units
	Maximum synaptic weight	2.0	arbitrary units
**Plasticity and Modulation**			
	Cholinergic gating window	150	ms
	Dopaminergic signal delay	300	ms
	Eligibility trace decay (τ_{eli})	600	ms
	D1 Homeostatic LTD coefficient (c)	-2e-2	arbitrary units
	D2 Homeostatic LTD coefficient (c)	-1e-2	arbitrary units
**Q-Learning Baseline (for comparison)**			
	Exploration rate (ε)	0.1	-
	Learning rate (α)	0.1	-
	Initial Q-values	0	-
**Simulation and Analysis**			
	Number of independent simulations	50	-
	Gaussian smoothing (σ)	3000	ms

Table S2. Complete parameter set used in the model and analysis.

the indirect (D2) pathway uses weights between 0.05 and 2.0. Synaptic connections incorporate variable delays randomly selected between 1ms and 66.7ms (1/15 seconds), reflecting biological transmission delays. Network stability during learning is maintained through a maximum weight ceiling of 10.0 for all connections.

Learning Mechanisms

Our model implements a biologically-inspired learning system where dopaminergic reward signals integrate with cholinergic responsibility signals to enable precise temporal learning. When an action neuron fires, it triggers a cholinergic signal (ACh dip) in all SPNs contained in that channel, creating a 150ms window during which synaptic plasticity can occur. This cholinergic modulation ensures temporal alignment between synaptic updates and behaviorally relevant actions, following recent experimental evidence from Berke et al.[10].

The dopaminergic (DA) system provides binary reinforcement 300ms after actions, reflecting experimental findings on critical windows for DA's influence on synaptic plasticity[13]. This delay allows the network to associate actions with their outcomes appropriately. During reward, DA affects synaptic plasticity in both D1 and D2 SPNs in different ways, while punishment triggers both synaptic changes and brief activations in D2 SPNs, facilitating rapid learning of adverse outcomes.

This implementation provides a simple mechanism for credit assignment: only synapses in the channel responsible for the selected action can undergo plasticity, and only during a brief window following that action. The learning process occurs through a three-factor rule combining spike timing, dopamine, and the action-triggered plasticity window. For each synapse, eligibility traces E(t) track the history of pre- and post-synaptic activity:

$$E(t) = \sum f(t_j - t_i) \cdot \exp\left(-\frac{t - t_i}{\tau_{\text{eli}}}\right)$$

where t_j and t_i represent pre- and post-synaptic spike times, $f(\cdot)$ denotes the spike-timing-dependent plasticity kernel (as defined in[18]), and τ_{eli} = 600ms determines the trace decay time constant. These eligibility traces store potential synaptic changes until validated by cholinergic signals, with the subsequent dopamine signal determining the direction and magnitude of modification. The learning parameters differ between pathways: D1 SPN synapses show enhanced sensitivity to reward signals, while D2 SPN synapses respond more strongly to punishment signals, reflecting their distinct roles in action selection.

To prevent unbounded synaptic growth and ensure long-term stability, we implemented an additional homeostatic plasticity mechanism in the form of multiplicative postsynaptic LTD. This term is proportional to both the current synaptic weight and the average firing rate $\bar{y}$ of the postsynaptic neuron:

$$\Delta w = c \cdot w \cdot \bar{y}$$

where c is a small constant, typically negative to produce LTD. This form of LTD approximates biologically observed synaptic scaling mechanisms[25] and is conceptually related to theoretical models of weight normalization[26]. While this rule successfully stabilizes synaptic weights, it also eliminates previously potentiated connections during contingency reversals, highlighting a trade-off between stability and memory retention discussed in the main text.

Performance Evaluation

Network performance evaluation encompasses both the temporal precision and correctness of action selection. The system considers responses valid only after 200ms of stimulus onset, ensuring sufficient processing time while preventing premature responses. Action correctness assessment involves continuous monitoring of action neuron activity, with rewards (+1) granted for correct actions, punishments (-1) for incorrect ones, and no feedback for absence of response. In cases of multiple active action neurons, the system employs a conservative approach: trials receive punishment if any incorrect action occurs, while rewards require both the presence of the correct action and absence of incorrect ones.

Neural activity analysis spans both individual neuron and population levels throughout learning. We generate raster plots to track spike timing across all neural populations (input neurons, D1 and D2 SPNs), complemented by population firing rates derived from Gaussian kernel smoothing (σ = 3000ms) of spike patterns. This smoothing provides a continuous representation of neural activity and facilitates the identification of temporal patterns. The analysis tracks the timing of action execution relative to these neural activity patterns, enabling investigation of the relationship between neural dynamics and behavioral outputs.

Synaptic weight analysis follows the evolution of connection strengths between input neurons and SPN populations throughout training. By grouping weights according to their associated action channels, we analyze both within-channel and between-channel distributions, computing mean weights and their variances for each training epoch. This approach reveals how the network develops and maintains stimulus-specific response patterns.

Our analysis pipeline generates three key visualizations:

- Raster plots showing precise spike timing across all neural populations.
- Smoothed firing rates of D1 and D2 populations with marked action execution times.
- Temporal evolution plots of synaptic weights.

For comparative analysis, we evaluated our model against a standard Q-learning baseline implementing an ε-greedy policy for action selection. The Q-learning agent treats each stimulus-action pair as a single-state bandit problem, with updates given by

$$Q_{t+1}(a) = Q_t(a) + \alpha\,[r_t - Q_t(a)]$$

We used $\alpha = 0.1$ (learning rate) and $\varepsilon = 0.1$ (exploration rate) as default parameters. With ε-greedy selection, the agent chooses the highest-value action with probability 0.9 and explores randomly otherwise. Importantly, accuracy is reported using the greedy policy (argmax over Q-values) derived from the learned values, rather than the stochastic ε-greedy behavior during training. This evaluation explains why accuracy can reach 100% despite continued exploration during learning.

In this deterministic single-state context, the learning rate α is inconsequential for asymptotic greedy accuracy: once the optimal action is sampled, its value will exceed all others for any $\alpha > 0$, ensuring convergence to perfect accuracy. α only modulates the rate at which Q approaches the reward magnitude. To illustrate this principle, we varied α systematically. We also varied ε to demonstrate its impact on performance. In each case, we tested 10 values between 0.1 and 1.0 while holding the other parameter constant at its default value. Learning curves track accuracy across training episodes, with 95% confidence intervals computed using the standard error of the mean across runs.

Code Availability and Reproducibility

Our implementation uses Python 3.9 with EDLUT simulator for neural simulations and NumPy for numerical computations. Data analysis and visualization rely on Matplotlib and Pandas. To ensure reproducibility, all random number generators were seeded for both network initialization and training procedures.

The complete implementation is available in our public GitHub repository: https://github.com/EduardoRosLab/STR_DA_ACh. This repository contains the full simulation codebase, configuration files with all model parameters, and analysis scripts. Users will find example notebooks demonstrating basic usage and with comprehensive installation instructions and a list of dependencies in the README.

Computational requirements vary with network size. Full experimental runs with 32 channels require approximately 1 hour on an 8-core processor with 32GB RAM, while smaller configurations (2-8 channels) complete within 1-30 minutes. The repository's README provides detailed instructions for environment setup and result reproduction.

References

1. Foster, N. N. *et al.* The mouse cortico–basal ganglia–thalamic network. *Nature* **598**, 188–194 (2021).
2. Hunnicutt, B. J. *et al.* A comprehensive excitatory input map of the striatum reveals novel functional organization. *elife* **5**, e19103 (2016).
3. Aosaki, T. *et al.* Responses of tonically active neurons in the primate's striatum undergo systematic changes during behavioral sensorimotor conditioning. *journal Neurosci.* **14**, 3969–3984 (1994).
4. Nougaret, S. & Ravel, S. Modulation of tonically active neurons of the monkey striatum by events carrying different force and reward information. *J. Neurosci.* **35**, 15214–15226 (2015).
5. Atallah, H. E., McCool, A. D., Howe, M. W. & Graybiel, A. M. Neurons in the ventral striatum exhibit cell-type-specific representations of outcome during learning. *Neuron* **82**, 1145–1156 (2014).
6. Duhne, M., Mohebi, A., Kim, K., Pelattini, L. & Berke, J. D. A mismatch between striatal cholinergic pauses and dopaminergic reward prediction errors. *Proc. Natl. Acad. Sci.* **121**, e2410828121 (2024).
7. Costa, K. M. *et al.* Dopamine and acetylcholine correlations in the nucleus accumbens depend on behavioral task states. *Curr. Biol.* **35**, 1400–1407 (2025).
8. Tubert, C., Paz, R. M., Stahl, A. M., Rela, L. & Murer, M. G. Striatal cholinergic interneuron pause response requires kv1 channels, is absent in dyskinetic mice, and is restored by dopamine d5 receptor inverse agonism. *bioRxiv* 2024–05 (2024).
9. Yamanaka, K. *et al.* Roles of centromedian parafascicular nuclei of thalamus and cholinergic interneurons in the dorsal striatum in associative learning of environmental events. *J. Neural Transm.* **125**, 501–513 (2018).
10. Berke, J. D. What does dopamine mean? *Nat. neuroscience* **21**, 787–793 (2018).

11. Dorst, M. C. *et al.* Polysynaptic inhibition between striatal cholinergic interneurons shapes their network activity patterns in a dopamine-dependent manner. *Nat. Commun.* **11**, 5113 (2020).

12. Da Silva, J. A., Tecuapetla, F., Paixão, V. & Costa, R. M. Dopamine neuron activity before action initiation gates and invigorates future movements. *Nature* **554**, 244–248 (2018).

13. Yagishita, S. *et al.* A critical time window for dopamine actions on the structural plasticity of dendritic spines. *Science* **345**, 1616–1620 (2014).

14. Reynolds, J. N. *et al.* Coincidence of cholinergic pauses, dopaminergic activation and depolarisation of spiny projection neurons drives synaptic plasticity in the striatum. *Nat. Commun.* **13**, 1296 (2022).

15. Urbanczik, R. & Senn, W. Reinforcement learning in populations of spiking neurons. *Nat. neuroscience* **12**, 250–252 (2009).

16. Calabresi, P., Picconi, B., Tozzi, A., Ghiglieri, V. & Di Filippo, M. Direct and indirect pathways of basal ganglia: a critical reappraisal. *Nat. neuroscience* **17**, 1022–1030 (2014).

17. Yin, H. H. *et al.* Dynamic reorganization of striatal circuits during the acquisition and consolidation of a skill. *Nat. neuroscience* **12**, 333–341 (2009).

18. González-Redondo, Á. *et al.* Reinforcement learning in a spiking neural model of striatum plasticity. *Neurocomputing* **548**, 126377 (2023).

19. Hjorth, J. J. *et al.* The microcircuits of striatum in silico. *Proc. Natl. Acad. Sci.* **117**, 9554–9565 (2020).

20. Gittis, A. H., Nelson, A. B., Thwin, M. T., Palop, J. J. & Kreitzer, A. C. Distinct roles of gabaergic interneurons in the regulation of striatal output pathways. *J. Neurosci.* **30**, 2223–2234 (2010).

21. Tepper, J. M., Tecuapetla, F., Koós, T. & Ibáñez-Sandoval, O. Heterogeneity and diversity of striatal gabaergic interneurons. *Front. neuroanatomy* **4**, 150 (2010).

22. Koós, T. & Tepper, J. M. Inhibitory control of neostriatal projection neurons by gabaergic interneurons. *Nat. neuroscience* **2**, 467–472 (1999).

23. Vogels, T. P., Sprekeler, H., Zenke, F., Clopath, C. & Gerstner, W. Inhibitory plasticity balances excitation and inhibition in sensory pathways and memory networks. *Science* **334**, 1569–1573 (2011).

24. Burke, D. A., Rotstein, H. G. & Alvarez, V. A. Striatal local circuitry: a new framework for lateral inhibition. *Neuron* **96**, 267–284 (2017).

25. Turrigiano, G. G. Homeostatic plasticity in neuronal networks: the more things change, the more they stay the same. *Trends neurosciences* **22**, 221–227 (1999).

26. Oja, E. Simplified neuron model as a principal component analyzer. *J. mathematical biology* **15**, 267–273 (1982).

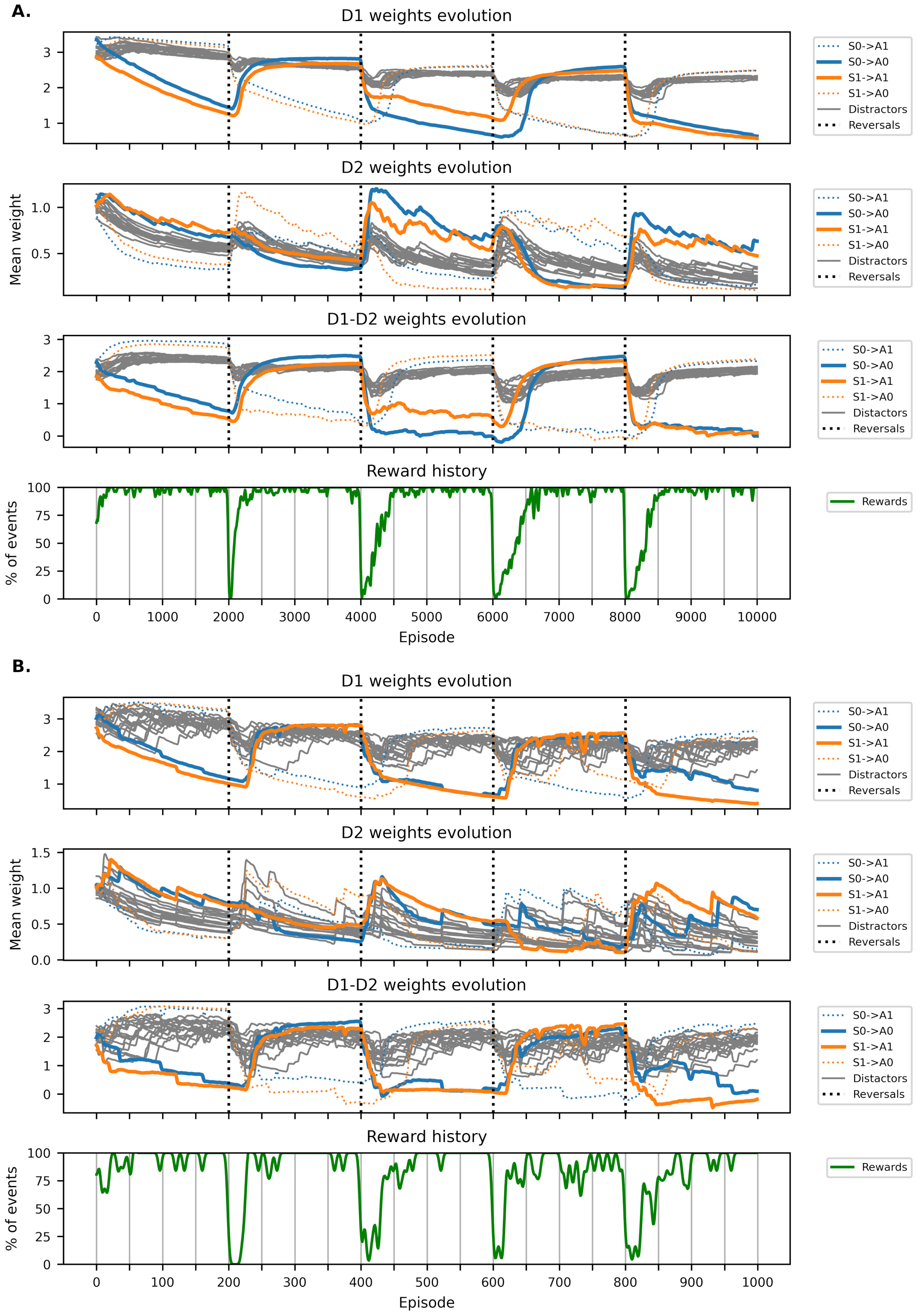

Figure S1. Effect of learning rate on acquisition speed and dynamics. Comparison of learning dynamics with standard learning rate (1e-3, top) versus increased rate (1e-2, bottom). While the faster learning rate reduces convergence time from 500 to 100 episodes per reversal, it introduces substantial noise in both synaptic weights and reward acquisition, obscuring the clear D1/D2 pathway dynamics visible at slower learning rates. Both simulations used identical architecture, parameters and task except for the learning rate constant and the time between reversals.

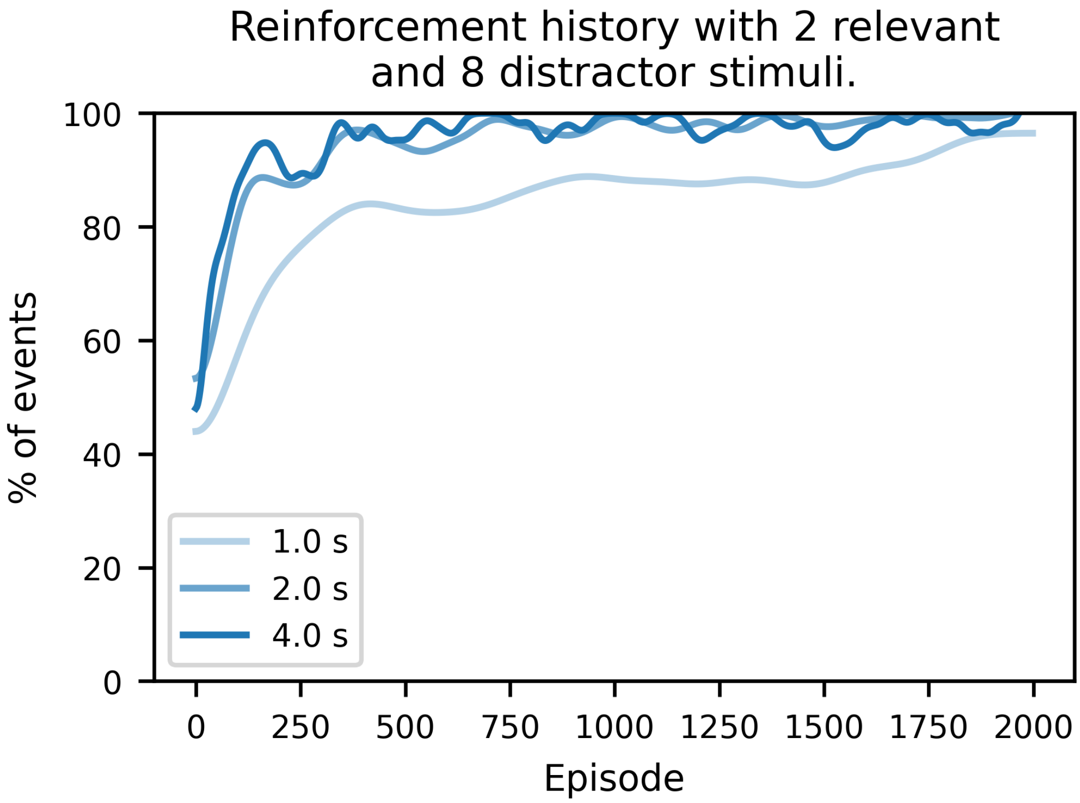

Figure S2. Effect of trial duration on learning performance. Mean accuracy over training episodes for trial durations of 1, 2, and 4 seconds. Shaded regions represent 95% confidence intervals across 5 random seeds. All conditions exhibit successful learning, with slightly slower convergence observed for 1-second trials. Performance for 2- and 4-second trials is indistinguishable, indicating robust learning across a broad range of temporal scales.

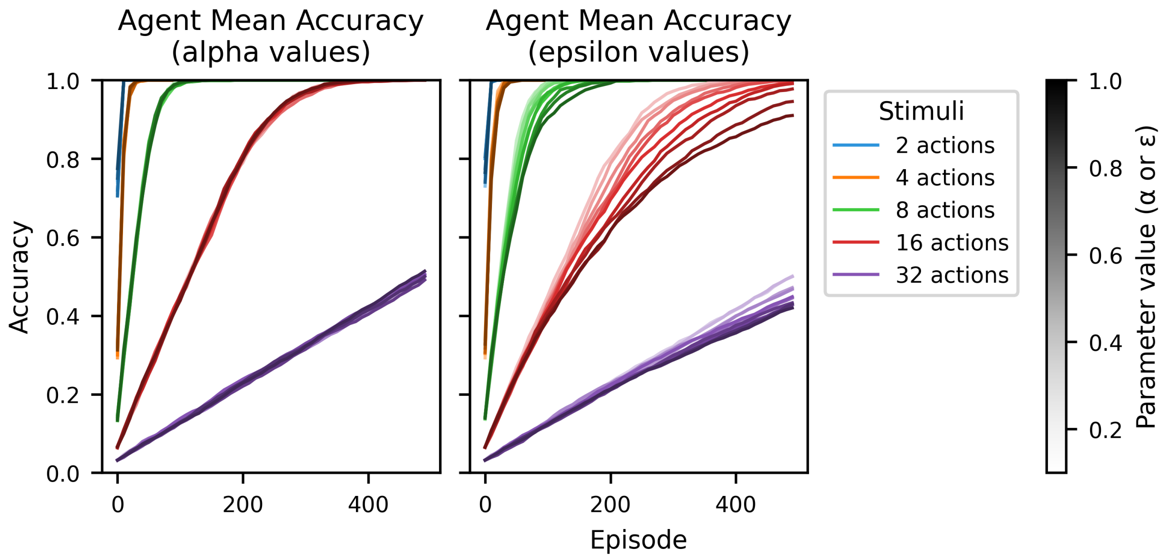

Figure S3. **Q-learning performance across different parameter values.** Parameter sensitivity analysis showing mean accuracy over 500 episodes for different values of (A) learning rate α and (B) exploration rate ε. Each panel shows results for tasks with 2, 4, 8, 16, and 32 stimulus–action pairs. For each parameter, we tested 10 values between 0.1 and 1.0 while holding the other constant at its default value ($\alpha = 0.1$, $\varepsilon = 0.1$). Shaded colors represent different parameter values, with lighter to darker shades corresponding to increasing α or ε (see grayscale bar). Accuracy is evaluated using the greedy policy (argmax Q), not the ε-greedy behavior during training, which explains why accuracy can reach 100% even when $\varepsilon > 0$. As expected, varying α does not affect asymptotic greedy accuracy but only the speed of convergence. In contrast, ε has a strong effect: higher values slow learning and reduce asymptotic accuracy. This analysis confirms that our baseline parameters provide consistent and non-pathological performance across task complexities.
